# MS-YOLOv8: multi-scale adaptive recognition and counting model for peanut seedlings under salt-alkali stress from remote sensing

**DOI:** 10.3389/fpls.2024.1434968

**Published:** 2024-11-07

**Authors:** Fan Zhang, Longgang Zhao, Dongwei Wang, Jiasheng Wang, Igor Smirnov, Juan Li

**Affiliations:** ^1^ College of Mechanical and Electrical Engineering, Qingdao Agricultural University, Qingdao, China; ^2^ College of Grassland Science, Qingdao Agricultural University, Qingdao, China; ^3^ High-efficiency Agricultural Technology Industry Research Institute of Saline and Alkaline Land, Qingdao Agricultural University, Dongying, China; ^4^ Department of Technologies and Machines for Horticulture, Viticulture and Nursery, Federal Scientific Agroengineering Center VIM, Moscow, Russia

**Keywords:** seedling rate, unmanned aerial vehicle (UAV), object detection, multi-scale, saline-alkali stress

## Abstract

**Introduction:**

The emergence rate of crop seedlings is an important indicator for variety selection, evaluation, field management, and yield prediction. To address the low recognition accuracy caused by the uneven size and varying growth conditions of crop seedlings under salt-alkali stress, this research proposes a peanut seedling recognition model, MS-YOLOv8.

**Methods:**

This research employs close-range remote sensing from unmanned aerial vehicles (UAVs) to rapidly recognize and count peanut seedlings. First, a lightweight adaptive feature fusion module (called MSModule) is constructed, which groups the channels of input feature maps and feeds them into different convolutional layers for multi-scale feature extraction. Additionally, the module automatically adjusts the channel weights of each group based on their contribution, improving the feature fusion effect. Second, the neck network structure is reconstructed to enhance recognition capabilities for small objects, and the MPDIoU loss function is introduced to effectively optimize the detection boxes for seedlings with scattered branch growth.

**Results:**

Experimental results demonstrate that the proposed MS-YOLOv8 model achieves an AP50 of 97.5% for peanut seedling detection, which is 12.9%, 9.8%, 4.7%, 5.0%, 11.2%, 5.0%, and 3.6% higher than Faster R-CNN, EfficientDet, YOLOv5, YOLOv6, YOLOv7, YOLOv8, and RT-DETR, respectively.

**Discussion:**

This research provides valuable insights for crop recognition under extreme environmental stress and lays a theoretical foundation for the development of intelligent production equipment.

## 1 Introduction

Peanut is rich in functional components and has high nutritional value, providing various important nutrients for the human body (Liu et al., 2022b). It has demonstrated significant advantages and remarkable development potential in the international edible oil supply (Tang et al., 2022). Peanut exhibits strong resistance, not only with drought tolerance, soil fertility adaptability, and the ability to grow in nutrient-poor conditions but also show certain tolerance to salt-alkali conditions (Huang et al., 2023). However, soil salinization and alkalization have become increasingly severe worldwide, and Salt-alkali stress has emerged as a pivotal factor constraining peanut production. Presently, saline-alkaline soils can be found in over 100 countries, encompassing a vast expanse of approximately 93.22 million hectares. Therefore, cultivating peanut varieties with excellent salt-alkali tolerance and effectively utilizing salt-alkali land to grow peanuts on a large scale is of great significance in improving the utilization rate of global salt-alkali soil and promoting the sustainable development of global agriculture.

As we all know, the emergence rate of seedlings is an important indicator to evaluate the salt-alkali tolerance of different crop varieties in the process of crop breeding. To calculate the emergence rate, it is necessary to count the number of seedlings. Traditional methods for calculating crop emergence rate rely heavily on manual counting in the field, which is time-consuming and lacks timeliness (Li et al., 2022b). Additionally, manual counting in the field often leads to the trampling of seedlings, resulting in damage or even death of the seedlings. To address these problems, the use of close-range remote sensing by unmanned aerial vehicle (UAV) for crop recognition and counting has emerged as one of the ongoing areas of active research focus (Xie and Yang, 2020).

In the field of crop recognition and counting using close-range remote sensing by UAV, many scholars have conducted early exploratory research. For example, Zhang and Zhao (2023) proposed a method for detecting and counting soybean seedlings based on the Otsu threshold algorithm. Li et al. (2019) used UAV to acquire high-resolution RGB orthophotos of potato seedlings. Otsu thresholding algorithm and Excess green index are used to extract potato seedlings from the soil. Then, the morphological features in the images are calculated as inputs to the random forest classifier, which is used to estimate the potato emergence rate. Banerjee et al. (2021) present a method to estimate the count of wheat seedlings at the seedling stage by utilizing multispectral images obtained from UAV. These studies, together with other related studies, have achieved good results by using image processing techniques or traditional machine learning models for seedling counting. However, these methods require manual feature extraction and threshold setting, which rely on strong professional expertise. Moreover, the performance of these methods may degrade in the presence of image data containing noise or outliers.

Some scholars use deep learning to solve the problems described above in traditional machine learning, especially in the areas of recognition, detection, and counting tasks (Xiong et al., 2024; Wang et al., 2022b; Li et al., 2023). Especially in agriculture, deep learning has been extensively employed in fruit defect detection (Zhu et al., 2023a; Xu et al., 2022b; Agarla et al., 2023), plant disease recognition (Haridasan et al., 2022; Xu et al., 2023a; Nawaz et al., 2024), seed analysis (Xu et al., 2022a; Zhao et al., 2023; Xu et al., 2023b), and many other aspects.

In the domain of seedling recognition and counting using close-range remote sensing by UAV combined with deep learning, Feng et al. (2020) used RGB images of cotton seedlings captured by UAV and the ResNet-18 to estimate the number of stands. Liu et al. (2022a) employ a Faster R-CNN model combined with the VGG16 network to recognize and count corn seedlings and verify the robustness of the model by RGB images captured at different locations with varying pixel resolutions. Gao et al. (2022) proposed an improved YOLOv4 model for detecting the number of corn seedlings. Liu et al. (2023) developed a rapid estimation system for corn emergence based on the YOLOv3 model. This system utilizes RGB images obtained from UAV to recognize the number, position, and size of seedlings. These studies, along with other related research, have achieved promising results in seedling counting based on UAV image data. These methods typically involve offline calibration and stitching of images, followed by the generation of ortho mosaic images using specialized software. These processing steps require high-performance computers and dedicated software. Therefore, these methods limit the real-time ability and application scenarios of object detection.

To address the limitations of seedling recognition and counting based on close-range aerial imagery, some researchers have proposed methods that utilize videos captured by UAV for seedling counting (Shahid et al., 2024; Lin et al., 2022). Shahid et al. (2024) introduce a video-based tobacco seedling counting model that utilizes YOLOv7 for tobacco seedling detection and the SORT algorithm for tracking and counting. Lin et al. (2022) employ an improved version of YOLOv5 combined with Deepsort to count peanut seedlings in real-time. Currently, research on crop recognition using close-range aerial sensing videos is in its early stages. To our knowledge, we have not seen other related literature reports except the above two papers. However, Shahid et al. (2024) only consider the detection of tobacco seedlings but do not consider the influence of weeds on the detection accuracy, and did not involve the problem of model lightweight required in practical applications. Although Lin et al. (2022) consider the influence of weeds on detection, it does not solve the problem of detection accuracy reduction caused by crop size differences under complex environmental stress, nor does it consider the problem of adaptive extraction of object features.

When crops are subjected to saline-alkali stress, different varieties have different degrees of tolerance. The appearance of seedlings of stress-resistant varieties is no different from that of peanuts in normal soil, while seedlings of non-stress-resistant varieties may be thinner, with smaller leaves and a yellowish color. These factors are easy to be confused with the soil background, which brings some difficulties to the accurate detection.

Inspired by the aforementioned studies, this research investigates the problem of recognition and counting for peanut seedlings under salt-alkali stress using close-range remote sensing. It takes the multi-scale effects of weeds on object morphology into consideration and proposes a multi-scale adaptive peanut seedlings recognition and counting model. The proposed Multi-Scale - You Only Look Once version 8 (MS-YOLOv8) model can directly and simultaneously recognize peanut seedlings and weeds in videos, and perform separate counting for each. The main contributions of this work are as follows:

1. Construction of a multi-scale adaptive convolution module, Multi-Scale Module (MSModule), which is embedded into the backbone network to enable the backbone of the model to have multi-scale feature fusion ability.

2. An object detection model MS-YOLOv8 is proposed. This model reduces the parameter count while improving the recognition ability for small objects without compromising accuracy. Furthermore, this model also solves the issue that the width and height values of the predicted bounding box with the same aspect ratio are significantly different from those of the ground truth box, which leads to the model not being optimized effectively, thus improving the convergence speed and regression performance.

3. Comparative analysis of seven typical object detection models, which provides a basis for selecting different models based on their detection performance.

The subsequent sections of this paper are structured as follows: Section 2 introduces the experimental materials, and describes the proposed model in this paper. Section 3 presents the experimental process and analyzes the experimental results from different perspectives. Section 4 discusses some issues encountered during the research, and explores the limitations of the proposed model as well as future research directions. In Section 5, a summary of the research conducted in this paper is provided.

## 2 Materials and methods

### 2.1 Data set acquisition

The data of peanut seedlings are collected from the saline-alkali soil experimental field of Qingdao Agricultural University, which is located in Maotuo Village, Lijin County, Dongying City, Shandong Province, China. This batch of peanuts contains nine varieties and was planted on the coastal beach saline soil with a cultivated layer of saline soil of about 20 cm on May 13, 2023, and the data was collected on May 28, 2023.

**Figure 1:**
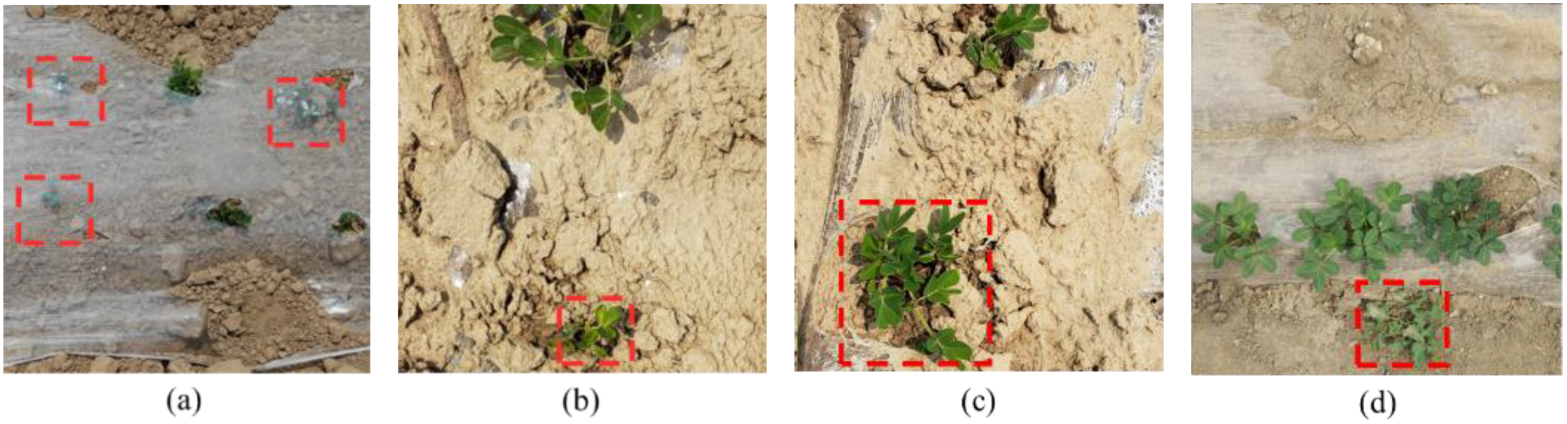
Examples of peanut seedlings images in different scenes. **(A)** Occluded by the plastic film; **(B)** Stunted peanut seedlings; **(C)** Uneven branching; **(D)** weed.

The aerial data was collected by DJI Mavic Air 2 UAV (DJI, Shenzhen, Guangdong, China). The dataset consists of 2696 images, and the image dataset is automatically divided into a training set, a validation set, and a test set in an 8:1:1 ratio using a Python script. The dataset including seedlings with weak growth and uneven branches blocked by plastic film, and weeds similar in appearance to peanut seedlings, as shown in **Figure 1**.

### 2.2 Proposed MS-YOLOv8 model

**Figure 2:**
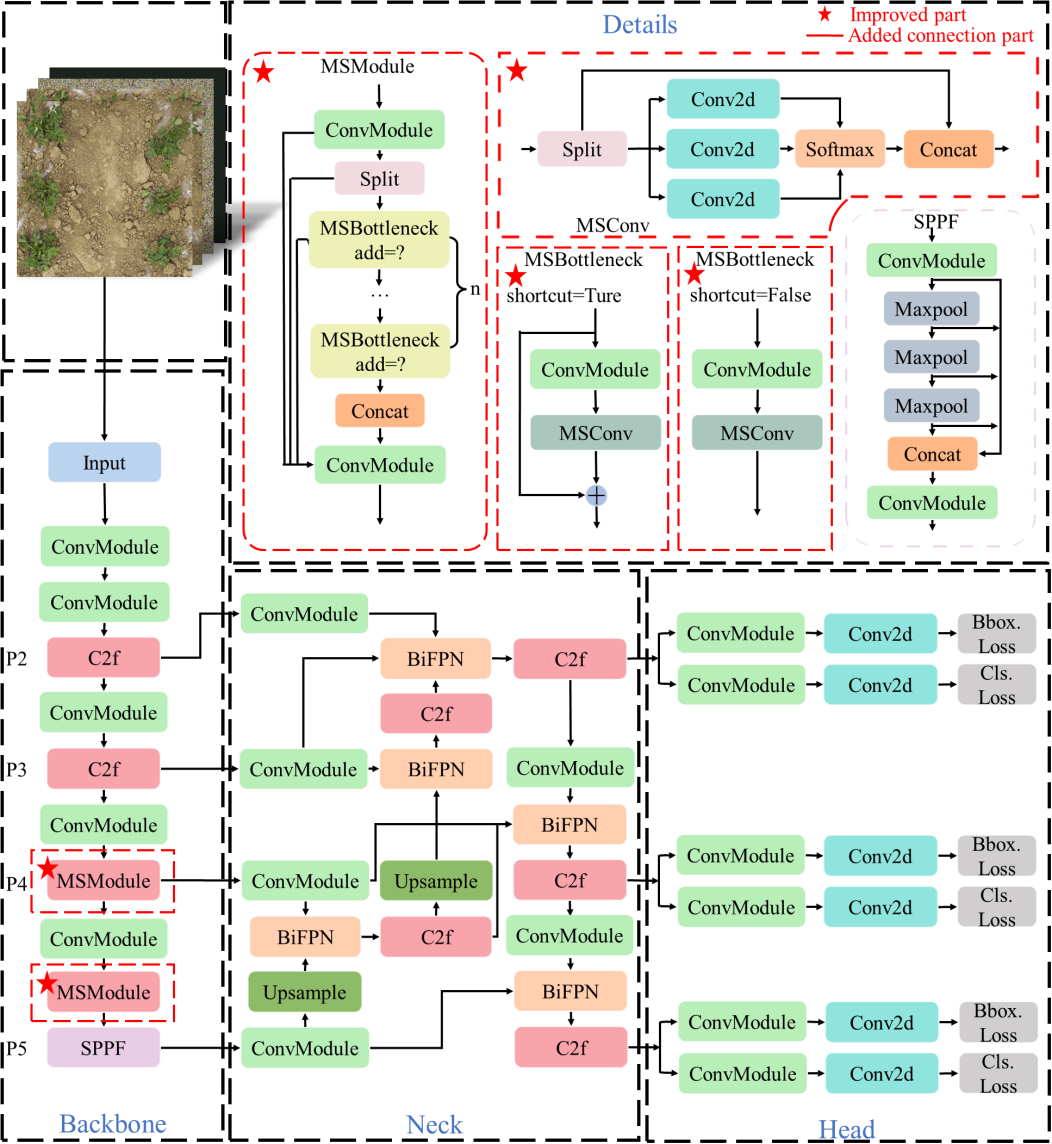
Structure of the proposed MS-YOLOv8 model.

In this paper, the lightweight model YOLOv8n (Reis et al., 2023) is selected. YOLOv8n is a lightweight parametric structure derived from the YOLOv8 model. To enhance the model’s performance and capacity for generalization, and to better adapt to the changes and challenges in actual scenarios, the MS-YOLOv8 model is proposed in this paper. Firstly, a multi-scale adaptive convolution module MSConv is designed and embedded into the C2f module to form a new feature extraction module, named MSModule. Embedding it into the backbone network can not only improve the multi-scale feature fusion ability of the model but also reduce the amount of calculation. Due to the dense density and different sizes of peanut seedlings, this paper introduces the Bidirectional Feature Pyramid Network (BiFPN) feature fusion method to improve the neck network used for feature fusion, and the 160 × 160 × 128 feature map of the P2 layer is fused to enhance the recognition effect of the model for small objects. In addition, to solve the problem of the model judgment of branches as single crops caused by long branches and the leaves are far from the main stem in some peanut seedlings, the MPDIoU loss function is used to enhance the bounding box regression effect and improve the detection performance of the model. The MS-YOLOv8 structure is shown in **Figure 2**, where the red dotted box marked with a red five-pointed star in the upper left corner is the modified or innovative part of this paper.

#### 2.2.1 Proposed module MSModule

In a convolutional neural network, each feature map is composed of a series of channels. Each channel represents a different feature that the layer of the network focuses on when extracting features from the input image, such as edges, textures, or shapes of objects. Generally, a larger number of channels can provide more feature information, but it also increases the computational and storage costs of the model. Moreover, there is a lot of redundant information between the feature maps output by each layer (Han et al., 2020), which also affects the training effect of the model.

**Figure 3:**
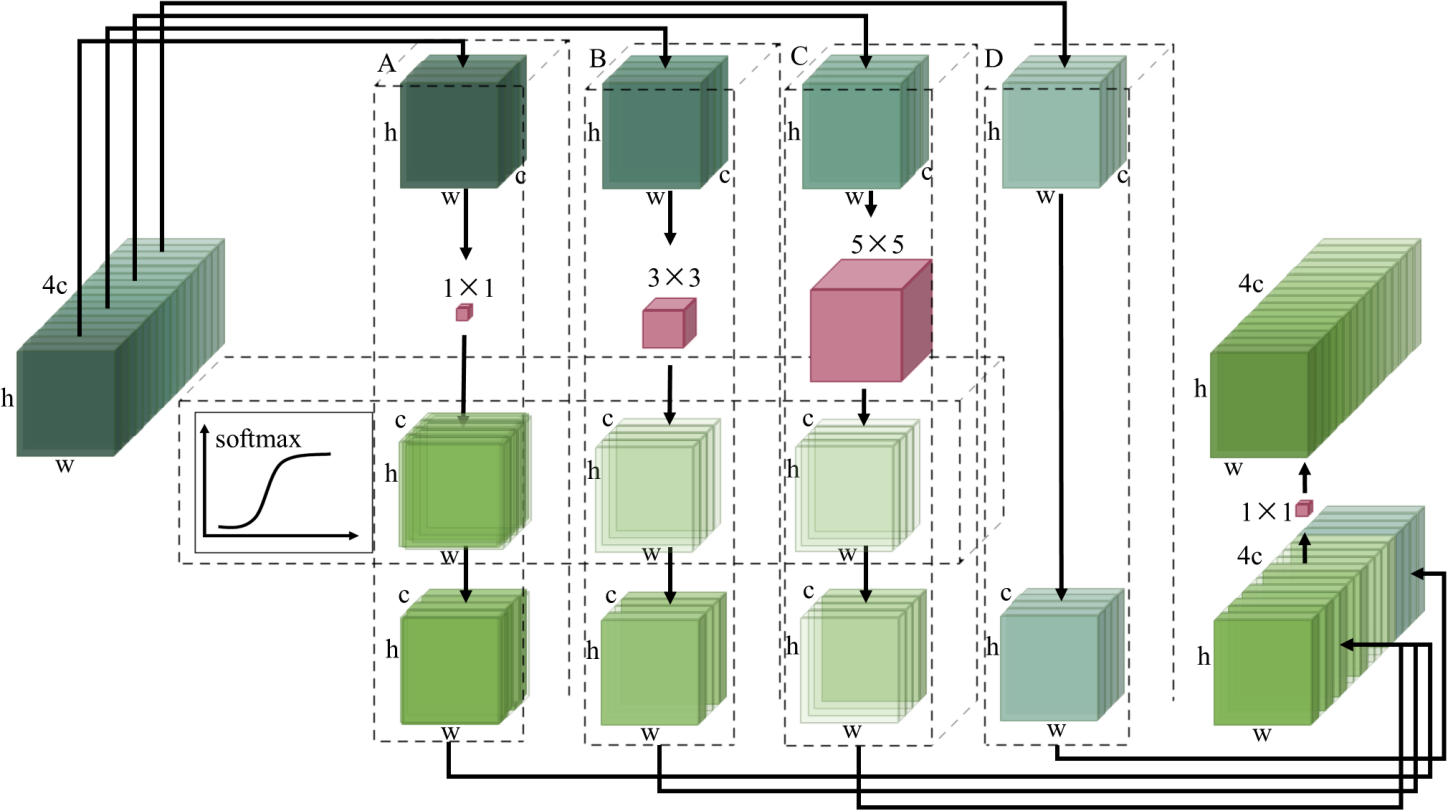
Structure of the MSConv module.

To realize lightweight multi-scale adaptive feature fusion, this paper designs a multi-scale adaptive convolution module MSConv, which makes the backbone network have the feature fusion effect. The structure of the MSConv module is shown in **Figure 3**.

In the schematic diagram of **Figure 3**, firstly, the input feature maps are divided into four groups A, B, C, and D in the channel dimension. The width and height of these four groups of feature maps are the same as the feature maps before grouping, and the number of channels is one-fourth of the original feature maps. The feature maps of group A, group B, and group C are passed through the convolution layer with 1 × 1, 3 × 3, and 5 × 5 size convolution kernels respectively, and the feature extraction operation is carried out.

Softmax function can adjust the output probability distribution according to the different input values. When the elements of the input vector have large differences, the softmax function will enhance these differences and make the probability distribution more obvious. The softmax function is used to automatically assign weights to each group of different channels to reflect their contribution to the network performance. The mathematical expression of the softmax function is as follows:


(1) $$\text{Softmax}(\epsilon_{i})=\frac{\text{exp(}\epsilon_{i})}{\sum_{j=1}^{N}\text{exp(}\epsilon_{i})}$$

where $\epsilon_{i}$ represents the first element in the input vector, and *N* is the dimension or length of the input vector.

By executing the softmax function on the channel dimensions of these feature graphs, the weight of interest for each channel can be obtained. These attention weights indicate how much each channel contributes to the final feature representation. A higher weight means that the channel is more important for the current input. By applying attention weight to each channel of the feature map, it can be weighted between feature maps at different scales. In this way, the important feature maps will have a greater influence on the final feature representation. Inspired by the idea of point-by-point convolution in the Mobilenetv2 (Sandler et al., 2018) model, the weighted feature map is concatenated with the original feature map. The channel information is exchanged through a convolution layer with a 1 × 1 convolution kernel to obtain the feature map after adaptive multi-scale feature fusion.

Then, the MSConv module is combined with the ordinary convolution module and the residual connection is introduced to form the MSBottleneck module. The residual structure of the MSBottleneck module directly transmits the input residual information by introducing residual connection and multi-layer convolution functions, making the gradient signal spread more smoothly.

Finally, the MSBottleneck module is embedded into the backbone network of YOLOv8, that is, the MSBottleneck module is used to replace the DarknetBottleneck module in the original C2f module to form a new feature extraction module MSModule.

#### 2.2.2 Improved neck network for enhancing feature fusion

**Figure 4:**
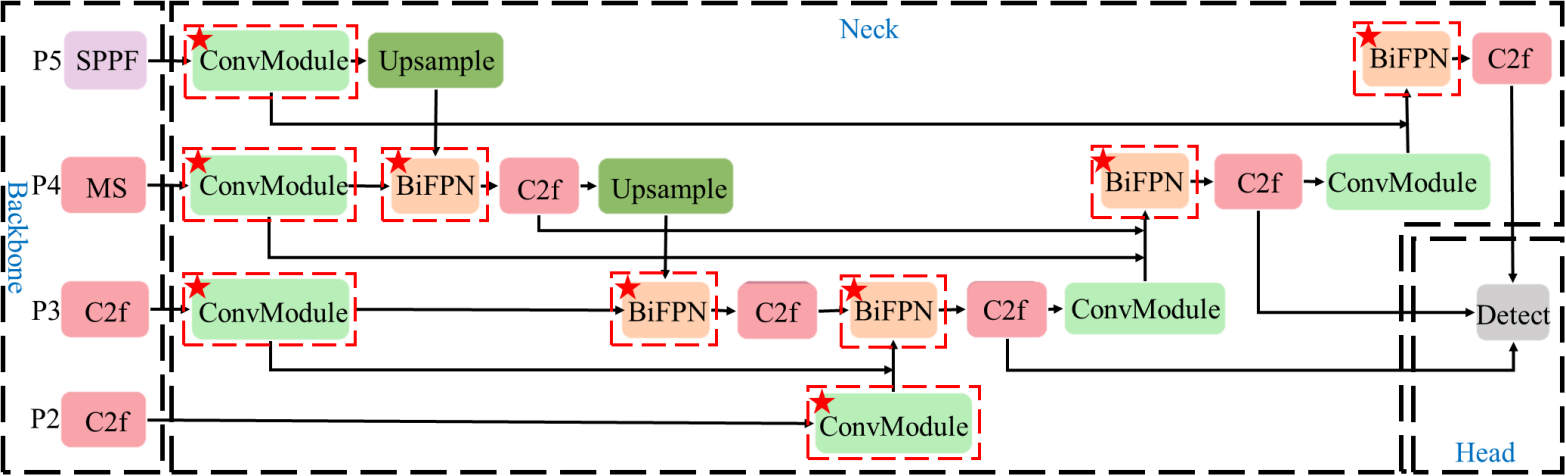
Structure of the improved neck network.

In this research, BiFPN is improved and used in the neck network of the YOLOv8 model. The neck network structure is shown in **Figure 4**.

As shown in **Figure 4**, P2-P5 represents the second to fifth layers in the feature pyramid structure of BiFPN, and the corresponding relationship with the convolution layer of the YOLOv8 backbone network is shown in the figure. The output feature maps of the P5, P4, and P3 layers of the feature pyramid structure are convolved and downsampled. The features are fused by bidirectional weighted fusion, and the detection heads of three scales are obtained. In particular, the feature map of the P4 layer is obtained by the feature extraction operation of MSModule designed in this paper. The size of these feature maps of different levels decreases gradually with the increase of the level, but the semantic information will increase. Finally, to enhance the detection effect of small objects, this research also uses the feature map extracted by the second layer C2f module, which is the P2 layer of the feature pyramid structure.

The process of BiFPN can be expressed as follows:


(2) $$F_{i}^{td}=Conv\left(\frac{\alpha_{1}\times F_{i}^{in}+\alpha_{2}\times R(F_{i+1}^{in})}{\alpha_{1}+\alpha_{1}+\beta }\right)$$

(3) $$F_{i}^{out}=Conv\left(\frac{\alpha_{1}^{\;'}\times F_{i}^{in}+\alpha_{2}^{\;'}\times F_{i}^{td}+\alpha_{3}^{\;'}\times R(F_{i-1}^{out})}{\alpha_{1}^{\;'}+\alpha_{2}^{\;'}+\alpha_{3}^{\;'}+\beta }\right)$$

where *R* stands for downsampling or upsampling operation, $F_{i}^{in}$ is the input feature at level *i*, $F_{i}^{td}$ is the intermediate feature at level *i*, $F_{i}^{out}$ is the output feature at level *i*. Additionally, *α* represents the parameter learned by the model, which determines the significance of various features in the process of feature fusion. Meanwhile, *β* is a predefined small value employed to prevent numerical instability.

The above improvements enable the network to effectively transmit and fuse the features from different levels. It can also realize bidirectional multi-scale feature fusion, enhance the fusion ability of deep and shallow feature information of images, and effectively deal with peanut seedlings and weeds of different sizes. And it provides fast detection speed while maintaining high accuracy.

#### 2.2.3 Improved loss function

To further enhance the precision and efficiency of bounding box regression while reducing computational overhead, the MPDIoU loss function (Siliang and Yong, 2023) is used in this paper. The MPDIoU loss function is a novel similarity measure for bounding boxes based on the minimum point distance. Its purpose is to address the limitation of existing loss functions, which fail to effectively optimize when the predicted bounding box and the ground truth box have the same aspect ratio but completely different width and height values. Simultaneously, it simplifies the computational process. The MPDIOU loss calculation process is as follows.

First, calculate the area of the intersection between the predicted bounding box and the ground truth box, as well as the sum of the areas of the two boxes. Then, use the difference between the area of the intersection and the sum of the areas to calculate the IoU. The calculation formula is as follows:


(4) $$IoU=\frac{X\cap Y}{X\cup Y}$$

where *X* and *Y* represent the area values of the predicted bounding box and the ground truth box, respectively.

Then, calculate the square of the distance between the upper-left point of the ground truth box and the predicted bounding box, and calculate the square of the distance between the lower-right point of the ground truth box and the predicted bounding box. It is calculated as follows:


(5) $$d_{1}^{2}={\left(x_{1}^{prd}-x_{1}^{gt}\right)}^{2}+{\left(y_{1}^{prd}-y_{1}^{gt}\right)}^{2}$$

(6) $$d_{2}^{2}={\left(x_{2}^{prd}-x_{2}^{gt}\right)}^{2}+{\left(y_{2}^{prd}-y_{2}^{gt}\right)}^{2}$$

where, $d_{1}$ represents the distance between the predicted bounding box and the top-left corner point of the ground truth box, and $d_{2}$ represents the distance between the predicted bounding box and the bottom-right corner point of the ground truth bounding box. $\left(x_{1}^{prd},y_{1}^{prd}\right)$ and $\left(x_{2}^{prd},y_{2}^{prd}\right)$ represents the coordinates of the upper left corner and lower right corner of the predicted bounding box, $\left(x_{1}^{gt},y_{1}^{gt}\right)$ and $\left(x_{2}^{gt},y_{2}^{gt}\right)$ represent the coordinates of the upper left corner and lower right corner of the ground truth box, respectively.

Finally, the normalized center distance and the difference in IoU score are used to calculate the value of MPDIoU, and the value of MPDIoU loss function is obtained by subtracting the value of MPDIoU from 1. It is calculated as follows:


(7) $$MPDIoU=IoU-\frac{d_{1}^{2}}{w^{2}+h^{2}}-\frac{d_{2}^{2}}{w^{2}+h^{2}}$$

(8) $$L_{MPDIoU}=1-MPDIoU$$

where $L_{MPDIoU}$ represents the *MPDIoU* loss function, *w* represents the width of the image, and *h* represents the height of the image.

The current YOLOv8 model utilizes the CIoU loss function (Zheng et al., 2019), which only considers the distance and size between the predicted bounding box and the ground truth box. However, most existing bounding box regression loss functions fail to optimize when the predicted box and the ground truth box have the same aspect ratio but significantly different width and height values. Therefore, this paper uses a more efficient MPDIoU loss function to replace the CIoU loss function in the original model, which not only improves the convergence performance of the model but also improves the effect of bounding box regression.

### 2.3 Real-time video object detection

**Figure 5:**
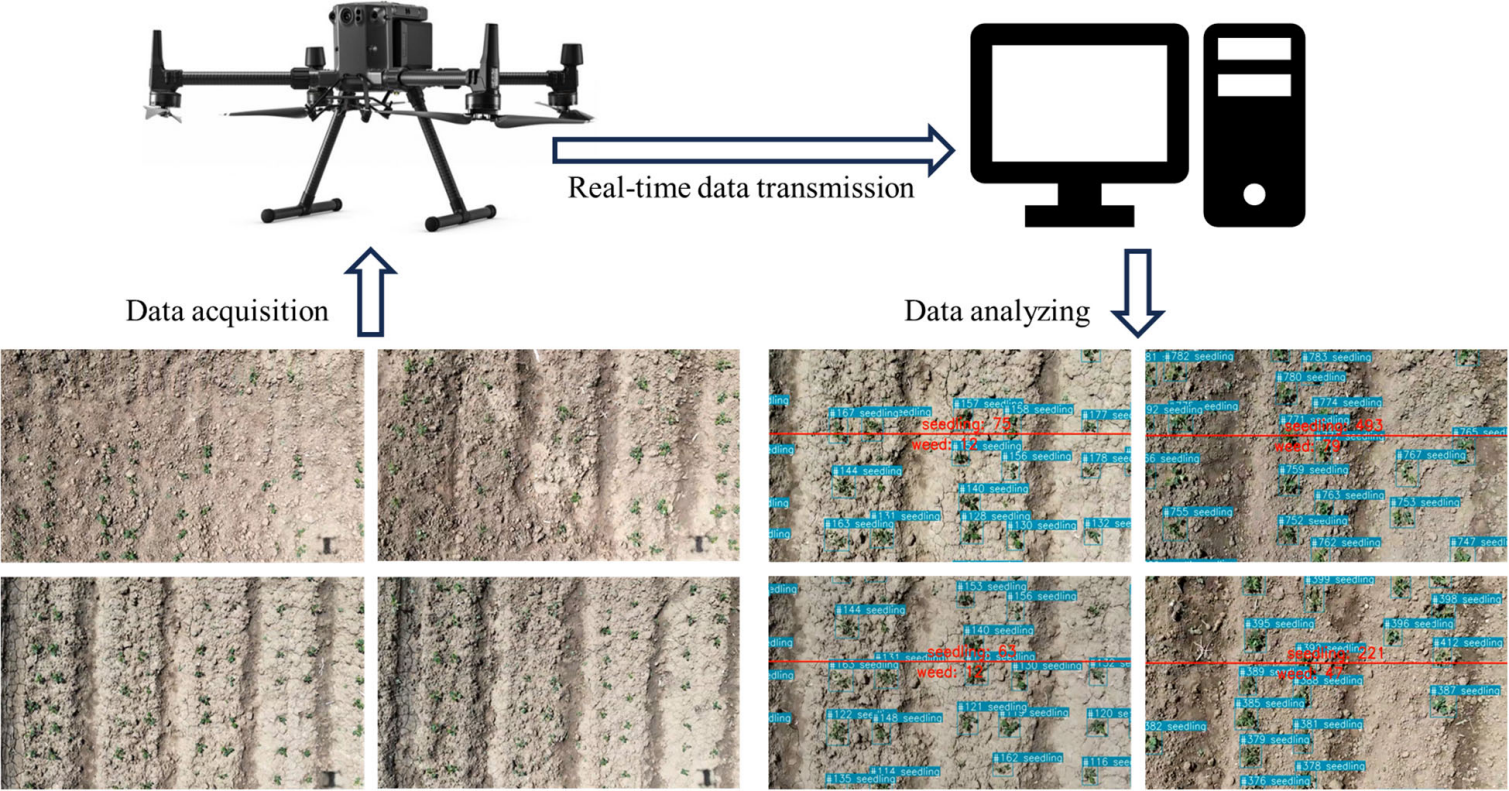
Illustration of real-time video object detection.

The model is based on the BoT-SORT tracking model built in the YOLOv8 model, enabling real-time counting of peanut seedlings and weeds. The specific procedure is as follows:

First, configure the NGINX server and RTMP module, start the RTMP server, and set up the RTMP address of the UAV to stream the footage to the server. Then, import the necessary libraries such as OpenCV and FFmpeg in the script, and use the VideoCapture class to read the real-time video from the RTMP stream. Within the loop of video frames, read each frame and draw a reference line in the center of the image. Utilize the MS-YOLOv8 model combined with the BoT-SORT model for object tracking, obtaining the ID and position information of the objects. Draw bounding boxes and labels for objects based on their type, and update the position history of the object. Finally, based on the position relationship of the objects with the reference line, continuously update the count of seedlings and weeds crossing the line, displaying the count information on the image. The marked frame images are displayed and written into a video file. The process is illustrated in **Figure 5**.

## 3 Experimental results and analysis

### 3.1 Evaluation index of the model performance

This research evaluates the performance of the object detection model using the following performance evaluation metrics: precision (*P*), recall (*R*), F1 score (*F*1), Average Precision (*AP*), mean Average Precision (*mAP*), and Frames Per Second (*FPS*). The calculation formulas for these performance metrics are as follows:


(9) $$P=\frac{TP}{TP+FP}$$

(10) $$R=\frac{TP}{TP+FN}$$

(11) $$F1=\frac{2TP}{2TP+FP+FN}$$

(12) $$AP=\int_{0}^{1}P(R)\text{d}R$$

(13) $$mAP=\frac{\sum_{\text{n}=1}^{N}AP(n)}{N}$$

(14) $$FPS=\frac{1000\text{ms}}{T_{pre}+T_{inf}+T_{pos}}$$

where TP represents the number of instances correctly detected as peanut seedlings or weeds by the model. FP represents the number of instances incorrectly detected as peanut seedlings or weeds when they are actually non-peanut seedlings or non-weeds (such as background or other objects). FN represents the number of instances incorrectly detected as non-peanut seedlings or non-weeds when they are actually peanut seedlings or weeds. The $T_{pre}$ represents the time for image preprocessing, including maintaining aspect ratio scaling and padding, channel transformation and dimensionality expansion, etc. The $T_{inf}$ represents the inference speed, which refers to the time required for an image to be preprocessed and output through a model. The $T_{pos}$ represents the post-processing time.

### 3.2 Experimental results

In this research, the improved YOLOv8 model is trained on a Windows 10 64-bit host, and all models are based on Python language. The deep learning model is built using the Pytorch2.10 framework and its built-in torch module. The processor is an Intel(R) Xeon(R) Silver 4316 CPU @ 2.30GHz. The graphics card is NVIDIA A40, and the deep learning framework is based on CUDA for GPU parallel acceleration.

**Figure 6:**
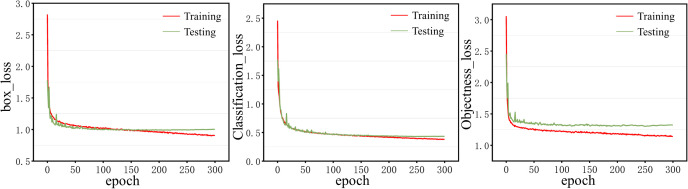
Training and Testing loss curves.

To facilitate better training of the model on the GPU, the batch size is set to 32, the image size is set to 1280, and the model is trained for 300 epochs. The remaining parameters are kept the same as the original parameters in the YOLOv8 model. The loss curve during training and testing is shown in **Figure 6**.

It can be seen from **Figure 6**, that the loss curves of box, object, and classification on the training set continue to decrease after using the above settings, which indicates that the model parameters are set appropriately and the model is trained well. **Figure 6** shows that the loss curves of boxes and objects on the testing set have converged after 150 epochs of training, and the loss curves of classification have converged after 250 epochs of training, indicating that 300 epochs of training can make the model fully learn. In addition, the loss curves of training and testing are close to each other, indicating that the model does not overfit.

**Figure 7:**
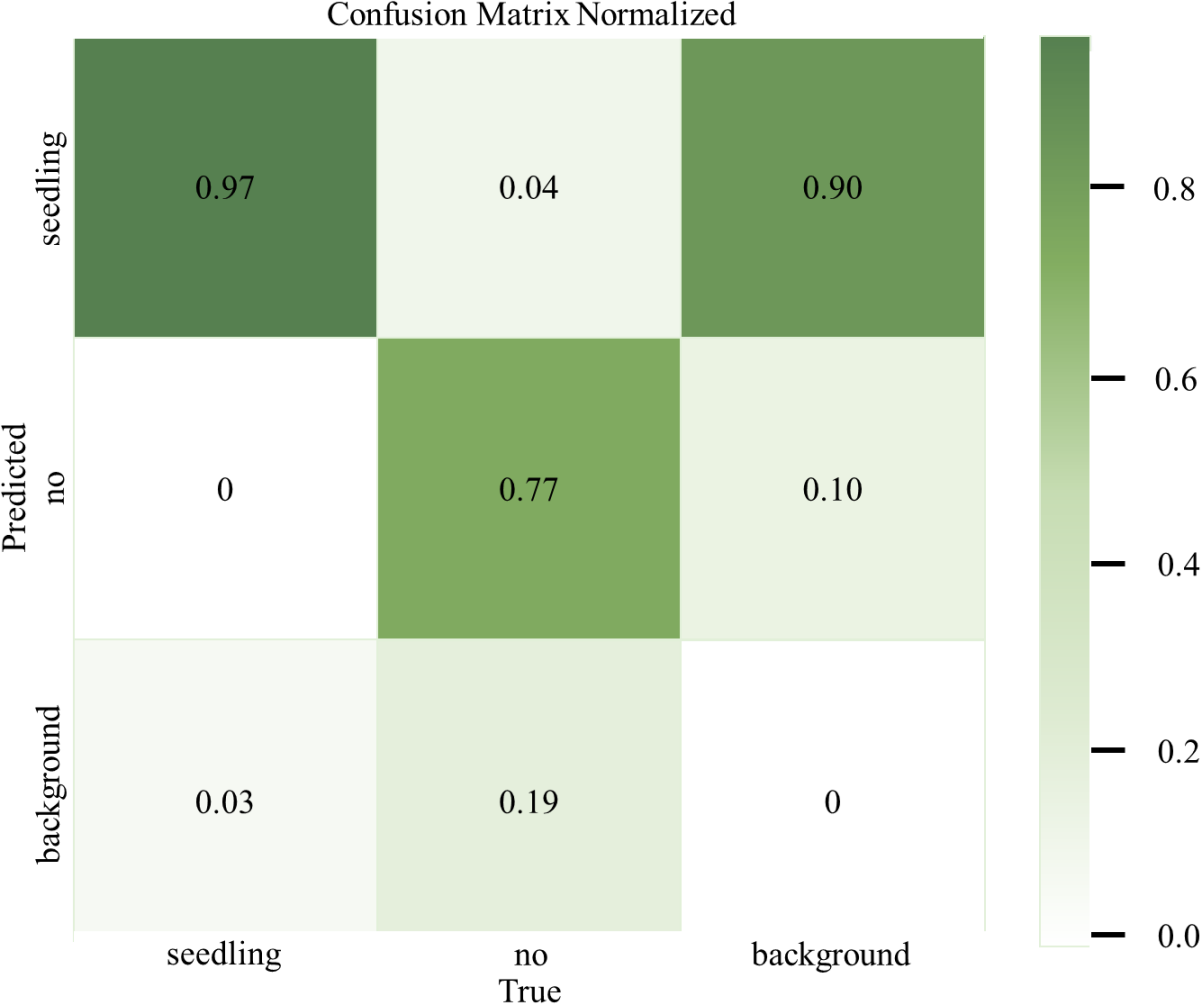
Confusion matrix of different categories.

To evaluate the actual effect of the MS-YOLOv8 peanut seedlings detection model in the recognition of various classes more intuitively, the confusion matrix of the MS-YOLOv8 model is plotted in this research, as shown in **Figure 7**.

From the confusion matrix, it can be observed that the MS-YOLOv8 model achieves an accuracy of 97% for peanut seedlings recognition and 77% for weeds recognition. The corresponding misclassification rates are 3% and 23%, respectively. This indicates that the MS-YOLOv8 model can accurately recognize peanut seedlings, but it exhibits a slightly higher misclassification rate for weeds. This could be attributed to the limited number of weed samples, which may have resulted in the model not being able to learn sufficient features for accurate weed classification.

**Figure 8:**
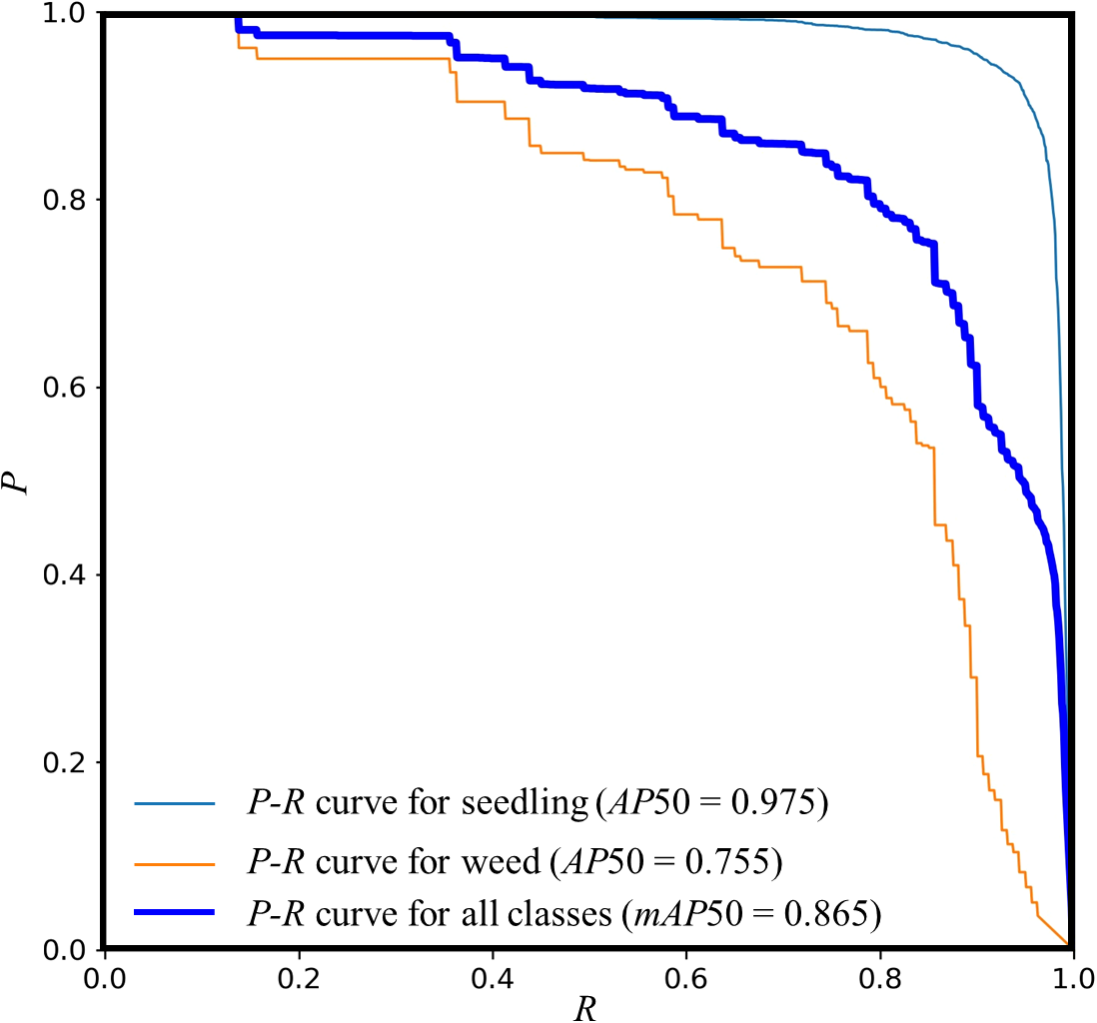
*P*-*R* curve of the MS-YOLOv8 model.

**Figure 8** illustrates the *P*-*R* curve of the MS-YOLOv8 model on the peanut seedlings dataset under salt-alkali stress. Experiments show that the MS-YOLOv8 model has high *AP* under different *R* levels. The *P*-*R* curve exhibits a smooth and upward-bending shape, indicating a good balance between *P* and *R*. The area under the *P*-*R* curve, known as the *AP*, is measured as 0.975 for peanut seedlings and 0.755 for weeds, resulting in an overall *mAP* of 0.865 when the IoU threshold is set to 0.5. Additionally, slight fluctuations in the *P*-*R* curve can be observed, which may be attributed to noise present in the dataset.

**Figure 9:**
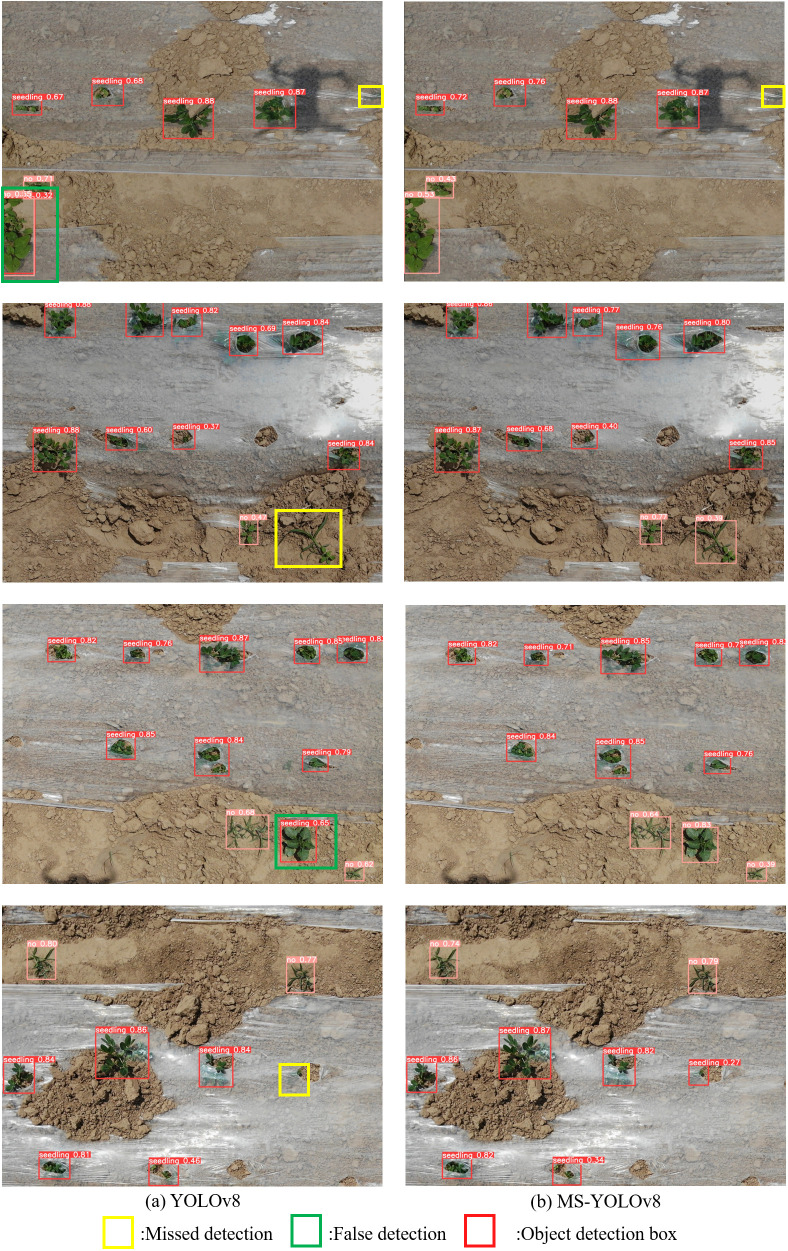
Comparison of actual detection effect between **(A)** YOLOv8 and **(B)** MS-YOLOv8.

After the improvements proposed in this paper, the MS-YOLOv8 model achieves more remarkable performance compared to the YOLOv8. **Figure 9** showcases the results of the object detection experiments on randomly selected images of peanut seedlings.

It can be seen from **Figure 9** that the YOLOv8 it easy to recognize weeds as peanut seedlings, resulting in missed detection. However, the MS-YOLOv8 not only has higher detection accuracy, but also improves the detection performance of small objects and partially occluded objects, but the improved model still has a phenomenon of missed detection of seedlings with serious occlusion.

### 3.3 Ablation experiment

To validate the detection performance of the proposed model and explore the impact of specific substructures on the model, this research conducted 8 ablation experiments based on the YOLOv8 model. The experimental results are shown in **Table 1**. (“+” indicates the introduced improvement, “-” indicates the non-introduction of improvement, and bold data indicates the optimal results in the experiments. Model 0 represents the YOLOv8 without any improvements).

**Table 1:** Result of ablation experiment.

Models	MSModule	BiFPN	MPDIoU	Class	*P* (%)	*R* (%)	*mAP*50 (%)	Params (M)
Model 0	–	–	–	All Seedling Weed	82.5 90.1 74.8	78.9 90.9 66.9	80.4 92.5 68.2	3.0
Model 1	+	–	–	All Seedling Weed	84.2 91.3 77.0	78.0 90.9 65.1	81.8 92.9 70.8	2.7
Model 2	–	+	–	All Seedling Weed	81.6 90.4 72.8	78.5 90.5 66.4	80.2 92.6 67.9	2.0
Model 3	–	–	+	All Seedling Weed	**84.7** 91.1 **78.3**	75.0 90.2 59.8	81.7 92.9 70.5	3.0
Model 4	+	+	–	All Seedling Weed	82.8 90.7 74.8	77.8 90.4 64.9	80.8 92.8 68.7	**1.9**
Model 5	+	–	+	All Seedling Weed	83.4 90.2 76.5	76.8 90.9 62.7	81.0 92.6 69.3	2.7
Model 6	–	+	+	All Seedling Weed	80.1 89.5 70.7	78.9 91.5 66.3	81.6 92.9 70.3	2.0
MS-YOLOv8 (Ours)	+	+	+	All Seedling Weed	81.3 **91.6** 71.0	**84.1** **94.8** **73.4**	**86.5** **97.5** **75.5**	**1.9**

Bold data indicates the optimal results in the experiments.

**Table 1** shows that the MS-YOLOv8 model outperforms the YOLOv8 model in terms of *R*, *mAP*50, and parameter size. The *R* and *mAP*50 have improved by 5.2% and 6.1% respectively, while the parameter size has decreased by 1.1M. When the BiFPN feature fusion structure, MPDIoU loss function, and MSModule are independently applied, the overall performance of the model is significantly improved.

Model 1, after introducing the MSModule, shows improvements of 1.7% in *P* and 1.4% in *mAP*50 compared to the YOLOv8. However, the *R* slightly decreases. This shows that the MSModule designed in this paper enhances the ability to recognize and classify the object by using a new feature extraction method, and improves the accuracy rate. This means that the model can more accurately distinguish the real object from the background or other categories, and reduce the case of false detection. However, due to the filtering operation in the added module, the model is more cautious in accepting the detection results, resulting in some real objects being missed and thus reducing the *R*.

**Figure 10:**
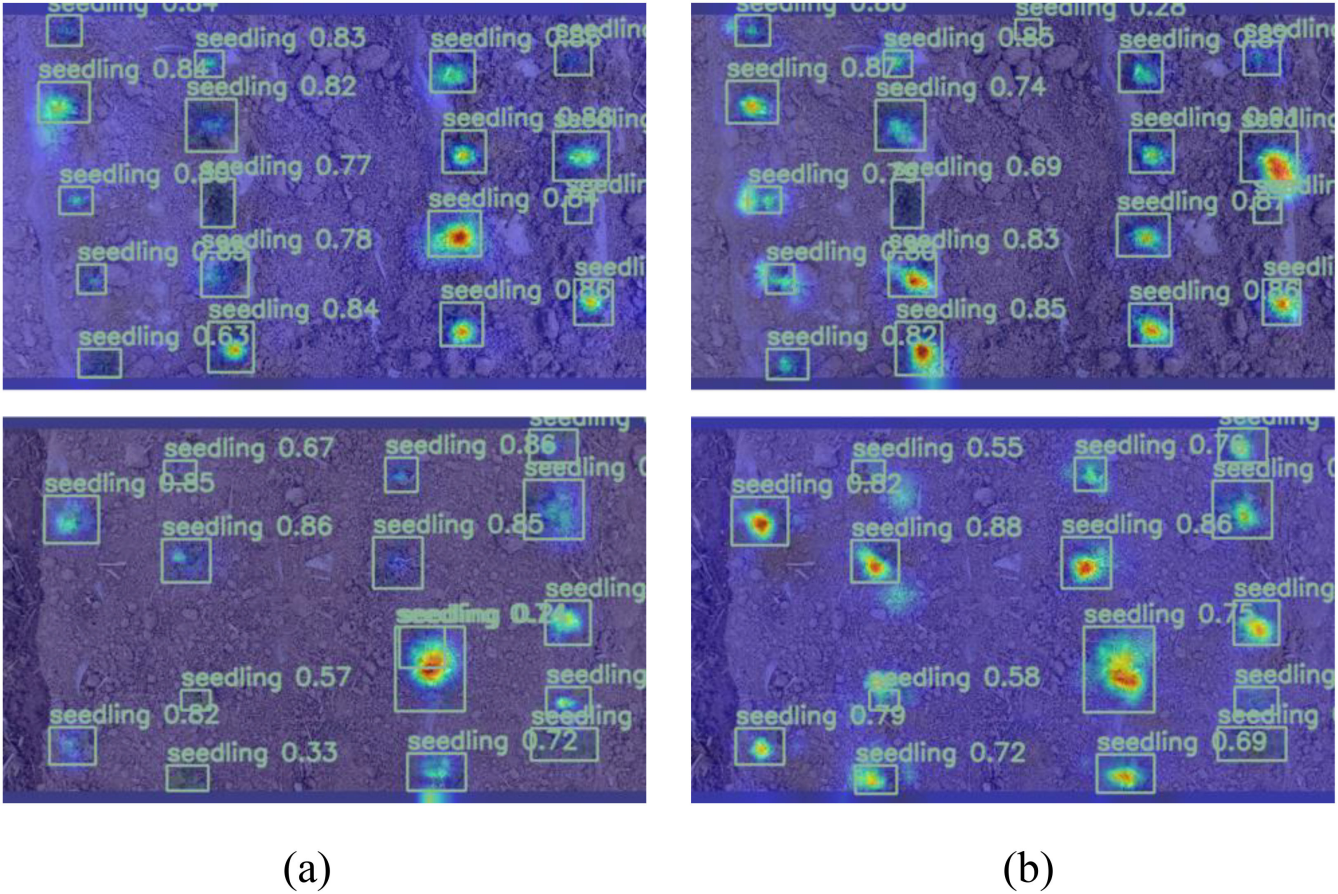
Heat map of the output feature map. **(A)** before improvement; **(B)** after improvement.

In this research, the thermal map (EigenGradCAM) technology was used to visually analyze the feature layer of Model 0 and Model 1 after adding MSModule, as shown in **Figure 10**. (Brighter colors indicate the areas that the model pays more attention to, while darker colors represent lower levels of attention.)

Since the subject of this research was peanut seedlings under saline-alkali stress, the size of the seedlings was different for different varieties. The before improvement network pays more attention to larger objects, while the after improvement network improves the attention to small objects.

A smaller convolution kernel excels at capturing intricate details and local features, whereas a larger kernel is adept at encompassing a broader scope of contextual information. Because MSModule uses the softmax function to adaptively select the proportion of feature maps extracted by convolution kernels of different sizes, the model can fully learn the characteristics of peanut seedlings with various appearances during training, instead of only focusing on easy to learn objects. Therefore, the addition of MSModule in this research is more conducive to the detection of peanut seedlings.

Model 2, after introducing the BiFPN feature fusion structure, shows improvements of 0.3% in *P* and 0.1% in *AP*50 for peanut seedlings recognition compared to the YOLOv8. Although the *R* decreases by 0.4%, the parameter size of the model is greatly reduced. This indicates that by introducing BiFPN in the neck network and redesigning the structure, it is possible to effectively retain feature information while reducing model parameters and computational complexity, thereby improving efficiency and performance.

Model 3, after introducing the MPDIoU loss function, also demonstrates satisfactory performance. It shows improvements of 1.0% in *P* and 0.4% in *AP*50 for peanut seedling recognition compared to the YOLOv8. Moreover, changing the loss function does not have any impact on the parameter size of the model.

The above results indicate that the adopted improvements are effective for model optimization. Model 4, by simultaneously introducing the MSModule and BiFPN feature fusion structure, *P* and *mAP*50 are improved by 0.3% and 0.4% respectively compared to the YOLOv8. Although the improvement in model detecting ability is not as significant as when the MSModule is used alone, the parameter size further decreases with the use of the BiFPN structure. Model 5 and Model 6 show good performance in *P* and *R*, respectively.

**Figure 11:**
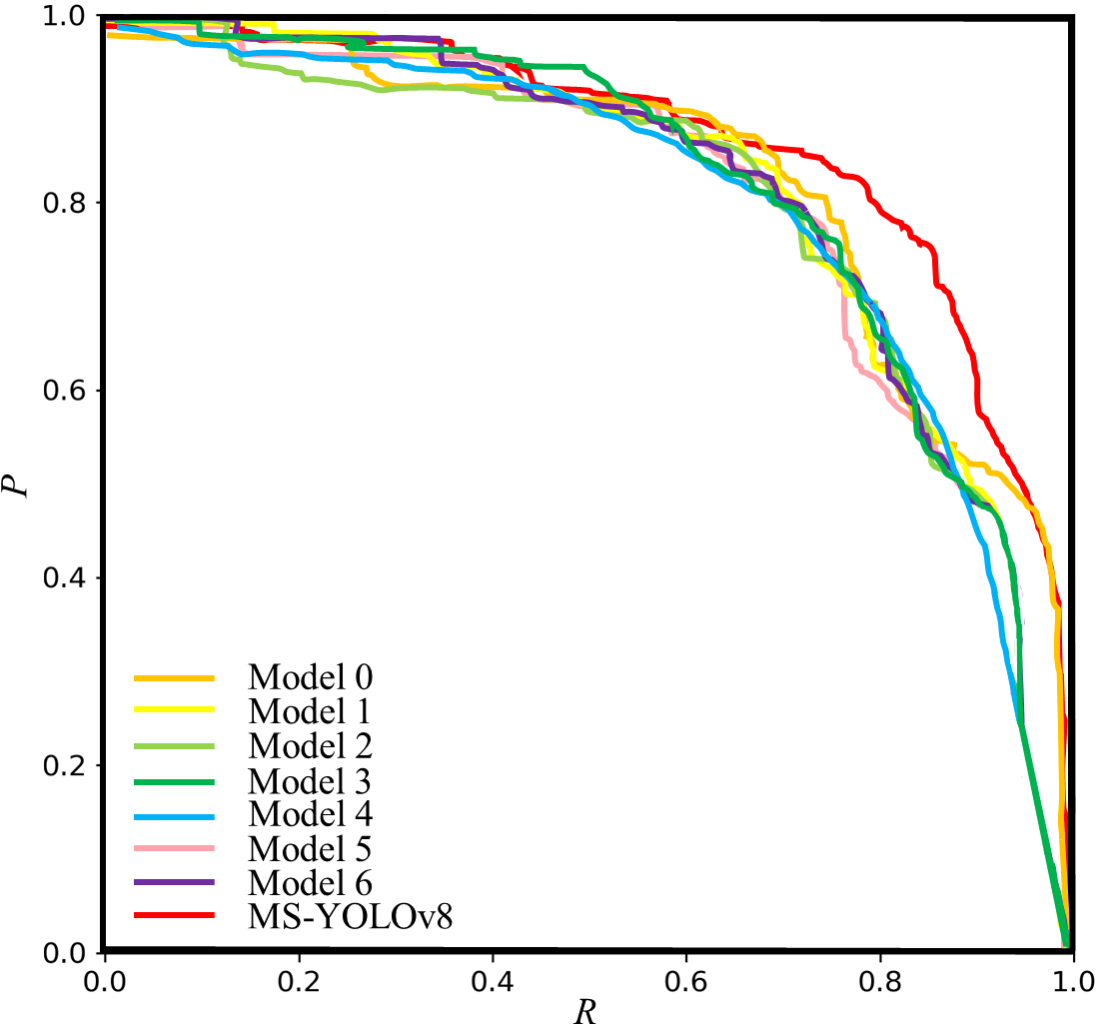
*P*-*R* curve for ablation experiment.

In this research, *P-R* curve of all models was drawn in **Figure 11**.

As shown in **Figure 11**, by comparing the *P*-*R* curves of different model architectures, it becomes evident that MS-YOLOv8 consistently exhibits superior performance compared to other model architectures across the entire range of *R*. In particular, the precision stays high when the recall is between 0.6 and 0.8. This indicates that the proposed model can effectively balance precision and recall under a specific threshold, which is suitable for scenarios with high requirements for both false positives and missed detections. The experimental results show that these modifications have a positive impact on balancing P and R.

Overall, the MS-YOLOv8 model proposed in this paper achieves the best results. Particularly, it shows improvements of 1.5% in *P*, 3.9% in *R*, and 5.0% in *AP*50 compared to the YOLOv8, with a significant reduction in parameter size. The parameter size of the model is reduced while enriching feature extraction by embedding the designed MSModule into the backbone network, using the BiFPN structure in the neck network, and replacing the loss function with the MPDIoU loss function.

### 3.4 Comparison with other models

In this section, a comparison is made among eight different object detection models: EfficientDet, Faster R-CNN, YOLOv5, YOLOv6, YOLOv7, YOLOv8, RT-DETR, and MS-YOLOv8, to validate the performance of the proposed model. The results are shown in **Table 2**. (Bold numbers in the table indicate the optimal performance achieved in the experiments.)

**Table 2:** Experiment comparison with different detection models.

Models	Class	*P* (%)	*R* (%)	*mAP*50 (%)	*mAP*50-95 (%)	*F*1 (%)	Params (M)	*FPS* (f/s)
Faster R-CNN (Ren et al., 2015)	All Seedling Weed	70.8 81.2 60.4	77.4 82.5 72.3	75.5 84.6 66.4	35.7 41.0 30.4	74.0 81.8 65.8	31.3	187.4
EfficientDet (Tan et al., 2019)	All Seedling Weed	82.1 86.5 **77.7**	78.6 87.1 70.1	80.3 87.7 72.9	51.2 59.2 43.2	80.3 86.8 73.7	4.1	215.4
YOLOv5 (Dong et al., 2022)	All Seedling Weed	79.3 89.5 69.1	80.3 91.3 69.2	81.2 92.8 69.6	53.3 70.0 36.7	79.8 90.4 69.1	2.5	322.6
YOLOv6 (Li et al., 2022a)	All Seedling Weed	81.2 90.1 72.3	80.0 91.4 68.6	80.7 92.5 68.9	53.2 69.9 36.5	80.6 90.7 70.4	4.2	312.5
YOLOv7 (Wang et al., 2022a)	All Seedling Weed	81.6 89.2 73.2	78.5 86.7 70.3	85.6 86.3 84.9	47.6 61.2 34.0	80.0 87.9 71.7	6.8	312.9
YOLOv8 (Reis et al., 2023)	All Seedling Weed	**82.5** 90.1 74.8	78.9 90.9 66.9	80.4 92.5 68.2	53.8 69.7 37.9	80.7 90.5 70.6	3.0	**333.3**
RT-DETR (Lv et al., 2023)	All Seedling Weed	72.5 82.1 62.9	**84.2** **95.5** 73.0	80.8 93.9 67.7	49.1 65.6 32.6	77.9 88.3 67.6	32.0	208.3
MS-YOLOv8(Ours)	All Seedling Weed	81.3 **91.6** 71.0	84.1 94.8 **73.4**	**86.5** **97.5** **75.5**	**56.8** **72.2** **41.3**	**82.6** **93.2** 72.2	**1.9**	272.2

Bold data indicates the optimal results in the experiments.

From **Table 2**, experimental results show that the MS-YOLOv8 is superior to the other seven models in *mAP*50 and *mAP*50-95 indicators. Although the detection speed and overall recognition accuracy of MS-YOLOv8 have slightly decreased compared to the previous version, it shows improvements in *R*, *mAP*50, *mAP*50-95, and *P* by 5.2%, 6.1%, 3.0%, and 1.5%, respectively. Moreover, the parameter size has decreased by 1.1M. Additionally, MS-YOLOv8 achieves the highest *F*1 score for peanut seedlings detection, with a value of 93.2, which is an increase of 2.7 compared to the YOLOv8. This indicates that MS-YOLOv8 achieves a good balance between *P* and *R*. In practical testing, the *FPS* for detecting a single image was 272.2, meeting the real-time recognition requirements for peanut seedlings under stress conditions. Furthermore, the advantage of MS-YOLOv8 is that it is a lightweight model with only 1.9MB, indicating relatively lower demands on storage and computational resources.

On the other hand, from **Table 2**, it can be seen that RT-DETR (Real-Time Detection Transform) achieves the highest *R*, indicating that it can better capture objects and detect them as much as possible, reducing the number of missed objects. A high *R* indicates good sensitivity of the model, effectively avoiding misclassifying true peanut seedlings as negative instances. However, its *P* is lower. This is because although RT-DETR performs well in detecting large objects by leveraging the global information brought by self-attention mechanisms, its performance in detecting small objects is relatively weaker, and it also has a lower real-time ability. Additionally, EfficientDet shows a higher *P* for weeds. This suggests although there is a smaller number of weed samples, this model can generalize better when dealing with categories with fewer samples and has better adaptability.

### 3.5 Experiment of counting peanut seedlings

Finally, the performance of the peanut seedling counting model proposed in this paper in the actual test is introduced. To evaluate the generalization ability of the model in field experiments, this research conducted experiments in two experimental fields, Test field 1 was saline soil, and Test field 2 was normal soil. The model was compared with the manual counting effect. The experimental results are shown in **Table 3**.

**Table 3:** Experiment results of manual and model.

	Counting type	Quantity	Rate (%)	Time (min)
Test field 1	Manual	689	100.0	20.0
	model	638	92.6	3.0
Test field 2	Manual	948	100.0	30.0
	model	940	99.1	4.5

Because the data collected in this research is diverse and contains peanut seedlings with different appearances and varieties. Therefore, the experimental results show that the model has strong generalization ability. The accuracy of the model in counting peanut seedlings in saline soil reaches 92.6%, and the accuracy of counting seedlings in normal soil was 99.1%.

Showing a significant advantage in terms of computation time compared to manual counting. The counting model based on videos is more convenient and easily applicable to practical crop cultivation compared to previous image-based counting models. The research results demonstrate that with the improvements made in this research, the model can detect peanut seedlings under salt stress more accurately and efficiently. The proposed model is practical and effective, and it has a positive impact on improving the emergence rate and breeding research.

## 4 Discussion

### 4.1 Performance analysis of MSModule

Lightweight convolutional neural networks are designed to achieve efficient inference with limited computational power (Li et al., 2021). These networks often use a series of optimization strategies to realize the lightweight, such as reducing network depth, minimizing the number of parameters, and using lightweight convolution operations. These lightweight designs allow models to run on lower hardware configurations. However, conventional lightweight models often suffer from a decrease in detection performance due to the reduction in parameters, resulting in the inability to capture complex object features and semantic information effectively. The proposed MSModule in this paper solves the decreased problem of detection performance effectively. The reasons of its effectiveness can be summarized as follows:

(1) This MSModule performs feature map grouping and feeds them into convolutional layers with different convolutional kernel sizes. This module enhances the ability of the model to select multi-scale features and improves feature extraction by adaptively adjusting the weights of feature map channels. (2) This module significantly enhances feature fusion by weighted information exchange between multi-scale feature maps. Additionally, the MSModule retains one-quarter of the input feature maps without any processing, which reduces redundant information and the number of parameters between feature maps, improving the inference speed of the model.

These structures of the MSModule enable it to learn and represent object features under limited computational power efficiently, allowing the object detection model to adapt to practical requirements better.

### 4.2 Detection effect analysis of model

Based on the YOLOv8 framework, MS-YOLOv8 demonstrates improved detection performance compared with YOLOv8, as shown in **Table 3**. The MS-YOLOv8 addresses some challenges for YOLOv8 to some extent, including missed detection, false detection, and imprecise bounding box regression. The reason may be summarized as follows:

(1) The designed MSModule uses convolution kernels of different sizes to extract multi-scale features of peanut seedlings and uses the softmax function to adjust the weight of different channels so that the feature channels with great contribution are paid more attention by the network. This enables MSModule to improve the feature selection ability and feature extraction ability and enables the model to distinguish peanut seedlings and weeds more accurately. (2) In this paper, the BiFPN feature fusion structure is introduced and the neck network is redesigned to enhance the feature fusion ability of the network. This enables the network to effectively fuse feature information at different levels and achieve more comprehensive semantic information transfer (Chen et al., 2024). (3) The MPDIoU loss function is used to improve the accuracy of the detection box and the ability to position the object precisely, as well as reduce false detections and missed detections of seedlings caused by the large phenotypic differences of peanuts under salt-alkali stress (He et al., 2024).

### 4.3 Cause analysis of missed and false detection

The MS-YOLOv8 model achieves 86.5% and 56.8% mAP50 and mAP50-95 scores, respectively, which are 6.1% and 3.0% higher than those of the YOLOv8 model. Although MS-YOLOv8 is better than YOLOv8 in detection effect, missed detections and false detections still occur. Several factors contribute to this problem:

(1) The used dataset is small, and the number of occluded and stunted peanut seedlings is limited, which makes the model unable to fully learn richer features, affecting the detection effect of the model (Wang et al., 2021). (2) The white translucent mulching film is easy to reflect light, and the recognition effect of the model is affected when the peanut seedlings are blocked by the mulching film. (3) The number of samples is unbalanced. The few and limited weed species in the field make the model unable to fully learn the characteristics of weed samples, resulting in the model sometimes misdetecting weeds with shapes similar to peanut seedlings (Li and Zhang, 2021). (4) In the process of model training, the improper setting of hyperparameters can prevent effective learning of object characteristics and contextual information. Therefore, this may cause the model to fail to detect the peanut seedlings.

### 4.4 Future work

Future work will first focus on enhancing the feature learning ability of the model and reducing the probabilities of false detection and missed detection. First, the semantic segmentation method (Zhu et al., 2023b) can be extended to use for the detection of peanut seedlings based on the existing work. Second, the propagation path of the feature layer or the structure of the convolutional module can be optimized to reduce the loss of the network when learning seedling features. In fact, this issue has been considered in some researches (Pang et al., 2022). Third, the structure of the whole model still has room for improvement, which has mentioned in some researches (Zhang et al., 2024).

## 5 Conclusions

To solve the problem of recognition difficulties caused by the existing models not considering the differences in size and shape of peanut seedlings under a saline-alkali stress environment, this paper proposes a peanut seedlings recognition and counting model MS-YOLOv8. The proposed approach improves the detection accuracy of peanut seedlings while maintaining a lightweight design. Experimental results demonstrate that the proposed MS-YOLOv8 model performs the peanut seedlings detection with the *AP*50 of 97.5%. Compared to typical models such as EfficientDet, Faster R-CNN, YOLOv5, YOLOv6, YOLOv7, YOLOv8, and RT-DETR, the MS-YOLOv8 achieves the highest detection accuracy with the minimum number of parameters. This research establishes a foundation for object recognition in extreme environments by close-range remote sensing. It provides a certain theoretical guidance for the development of an intelligent monitoring platform for peanut.

## Data Availability

The datasets presented in this study can be found in online repositories. The names of the repository/repositories and accession number(s) can be found below: https://github.com/zfvincent1997/MS-YOLOV8.
